# The incidence of and mortality from leukaemias in the UK: a general population-based study

**DOI:** 10.1186/1471-2407-9-252

**Published:** 2009-07-26

**Authors:** Fatima Bhayat, Emma Das-Gupta, Chris Smith, Tricia McKeever, Richard Hubbard

**Affiliations:** 1 Division of Epidemiology and Public Health, University of Nottingham, Clinical Sciences Building, Nottingham City Hospital, Hucknall Road, Nottingham, NG5 1PB, UK; 2 Division of Academic Haematology, Nottingham City Hospital, Hucknall Road, Nottingham, NG5 1PB, UK

## Abstract

**Background:**

The acute and chronic leukaemias constitute about 2.5% of all newly diagnosed malignancies and kill over 4000 people/year in the UK, yet there is little accurate up-to-date data on how the incidence of and mortality from leukaemias vary with socio-economic status in the UK. We aimed to quantify the incidence of and mortality from leukaemias in the UK and their variation with gender, age, year of diagnosis as well as socio-economic status.

**Methods:**

All incident cases of leukaemia were identified in 'The Health Improvement Network' (THIN) General Practice dataset. Crude incidence rates and incidence rate ratios (using Poisson Regression) stratified by age, gender, year of diagnosis and socio-economic status were calculated. Median survival and hazard ratios for risk of death (using Cox regression) were then calculated, and stratified in a similar manner.

**Results:**

A total of 4162 cases of leukaemia were identified, 2314 (56%) of whom were male. The overall incidence of leukaemia was 11.25 per 100 000 person-years. The age and gender distributions of ALL, AML, CLL and CML were similar to UK cancer registry data. The incidence of leukaemias was independent of socio-economic class. Median survival from leukaemia was 6.58 years and mortality increased with increasing age at diagnosis. The prognosis in AML was dismal and worsened with increasing socio-economic deprivation. For other leukaemias mortality was independent of socio-economic status.

**Conclusion:**

This is the first general population study to describe the incidence of and mortality from leukaemias in the UK by socio-economic status. Similar mortality across socio-economic gradients in the leukaemias studied suggests equal access to and uptake of services. The exception to this was in AML, where poorer survival in AML patients from lower socio-economic classes may represent a class bias in treatment offered and/or greater co-morbidity in these patients, and warrants further exploration.

## Background

The acute and chronic leukaemias constitute 2.5% of all cancers and together are the 12^th ^most common cancer registered in the UK [1]. Approximately 7000 people are diagnosed with these diseases and more than 4300 people die from leukaemias in the UK each year [1]. Although the Office of National Statistics, cancer charities and cancer registries in the UK make valuable contributions to our knowledge on its variation by gender and age, as well as trends over time, there is a paucity of contemporary data on disease incidence and mortality and how these vary by socio-economic circumstances. A source of general population derived figures that could be updated regularly would be very helpful when planning medical services and ensuring equal access to these services.

More than 97% of people in the UK are registered with a general practitioner, which makes GP databases an excellent general population source of data on disease incidence and mortality. Diagnoses of cancer have been examined in general practice databases before and have been found to be valid [2]. This means that computerised general practice data may be a valuable new resource for leukaemia research. In addition to medical and prescribed drug histories held in these datasets, such datasets can also readily supply controls for studying disease aetiology, which gives them an advantage over registry data.

As part of a programme of research on leukaemia using general practice datasets we set out to quantify the incidence of and mortality from leukaemias in the UK, and the variation of these with gender, age, calendar time and socio-economic status.

In this article we report the associations we found between socio-economic class and both leukaemia incidence, and mortality in the UK.

## Methods

'The Health Improvement Network' (THIN) dataset is a computerised dataset from over 330 general practices across England, Scotland, Wales and Northern Ireland, and includes 5.7 million patients, 2.5 million of whom are actively contributing data and can be prospectively followed. Data held include patient demographic data, Townsend score of socio-economic deprivation, as well as their medical and prescribed-drug histories. 'THIN' data represent all sections of the general population of the UK [3]. The total number of usable patients in the dataset was 5 395 612, with 2 592 133 actively contributing data on 1st July 2007 when data for this study were extracted. Data from 1987 to 2006 are included in this study.

A list of diagnostic codes, called READ codes (available on request) was used to identify all cases with an incident diagnosis of leukaemia in the dataset. Since retrospective diagnoses may be entered into patient records at the time the patient first joins a general practice, or when a general practice first starts to use diagnostic software, cases were only included as incident cases in the analyses if their first ever recording of a diagnosis of leukaemia occurred at least 12 months after their general practice records were computerised. We grouped our leukaemia diagnoses on the basis of READ code descriptions as follows: Acute Lymphoblastic Leukaemia (ALL), Chronic Lymphocytic Leukaemia (CLL), Unspecified Lymphocytic or Lymphoid Leukaemia, Acute Myeloid Leukaemia (AML), Chronic Myelogenous Leukaemia (CML), and Unspecified Myelogenous or Myeloid Leukaemia. The 'unspecified' groups do not represent discrete diseases, but represent patients with either lymphoid or myeloid leukaemia, but who we could not further classify into acute or chronic categories. Myelodysplastic Syndromes have been excluded. In order to calculate disease incidence we used the entire population within the THIN dataset as our denominator. The 'THIN' mid-year population (as at 1^st ^July) was stratified by gender, age and Townsend score for this purpose.

For our analysis we grouped age at diagnosis into 5, 20-year age categories with category 1 being those aged <20 yrs and category 5 representing those aged 80 yrs and above. Year of diagnosis was grouped into 4, 5-year bands with 1987–1991 being the first year-band and 2002–2006 the most recent. Townsend Score, a measure of socio-economic deprivation, is derived from 2001 census output data and is a multifaceted index of deprivation based on unemployment, car ownership, home ownership and overcrowding. Townsend Scores are divided into quintiles, with higher scores representing greater socio-economic deprivation [4]. Whilst not an individual measure of deprivation, it represents a small homogenous socio-geographic area of about 150 homes.

Initially we calculated crude incidence rates for leukaemia and its subtypes, and stratified these estimates by gender, age at diagnosis, year of diagnosis and Townsend Score. We used Poisson regression to estimate incidence rate ratios, both independently and mutually adjusted for gender, age, year of diagnosis and Townsend Score.

We estimated median survival from leukaemia and its subtypes, and then used Cox regression to calculate hazard ratios, adjusted for gender, age-category, year of diagnosis and Townsend Score. All analyses were conducted using STATAv9.

Ethical approval for the study was obtained from the Nottingham Research Ethics Committee.

## Results

### Incidence

**Figure 1:**
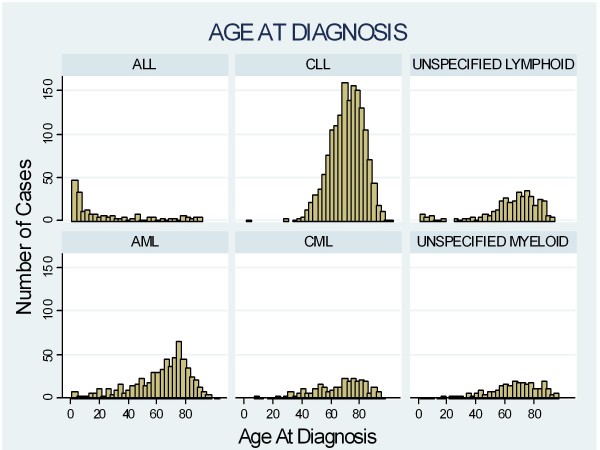
**Age at Diagnosis of leukaemia subtypes**.

We identified a total of 4162 cases of leukaemia, 2314 (56%) of whom were male. Children aged 10 or younger constituted 4.5% of cases, of whom 56% were male. The overall incidence of leukaemia in the study population was 11.25 per 100 000 person-years. We were able to identify 3226 (78%) of the leukaemia cases as falling into one of the 6 sub-types of interest. The crude incidence rates are shown in Additional file 1. CLL was the most common subtype and ALL the least common, with crude incidence rates of 4.20 and 0.49 per hundred thousand person years, respectively. The distribution of age at diagnosis of leukaemia by subtype is shown in Figure 1 (see Figure 1). Most cases of ALL were diagnosed in early childhood, whilst the other forms of leukaemia were largely diseases of adulthood that increased in incidence with age.

Results of Poisson regression are shown in Additional file 2, in which incident rate ratios are adjusted for all other variables in the table. Females had a lower incidence of CLL, unspecified lymphoid leukaemia and AML. The incidence of all sub-types, except ALL, increased with increasing age at diagnosis (p for trend < 0.001). Interestingly the incidence of ALL decreased with increasing socio-economic deprivation, although this trend did not reach statistical significance (p for trend = 0.15).

### Survival

**Figure 2:**
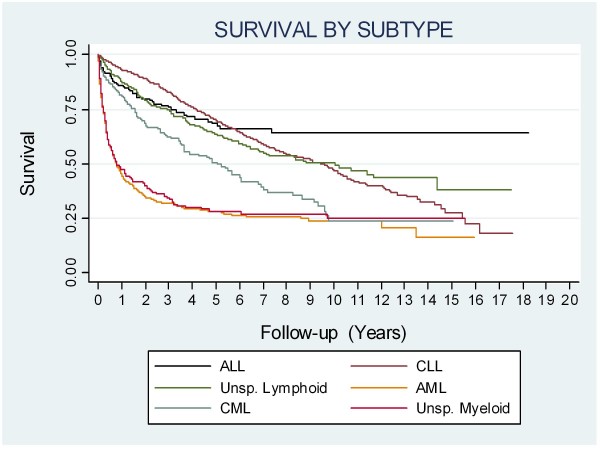
**Kaplan-Meier survival curves for each leukaemia subtype**.

The median survival from all leukaemias in this study was 6.58 years. The median survival from each subtype is shown in Additional file 3 and the Kaplan-Meier survival curves are plotted by subtype in Figure 2. ALL had the best prognosis, with more than 50% of cases surviving the follow-up period. For this reason we calculated the 5-year survival for ALL and this was 69%. The poorest median survival, of only 9 1/2 months was seen in AML.

The results of Cox regression are shown in Additional file 4. We found that women with CLL had a better prognosis than men (HR 0.58, p < 0.001, 95%, C.I. 0.48–0.71). These hazard ratios were adjusted for age-category, Townsend Score and year of diagnosis. We did not find this gender difference in the other specified leukaemia sub-types studied. We saw a poorer prognosis with increasing age at diagnosis in all subtypes (p for trend < 0.001). In AML the mortality increased with increasing deprivation such that mortality rates were nearly 50% higher in the most deprived quintile of Townsend Score than for the least deprived quintile (p for trend = 0.03). We did not find a socio-economic gradient in survival in other leukaemias. Mortality has remained stable over calendar time, except in CML, which showed a decreasing trend in mortality of borderline statistical significance (p for trend = 0.05).

## Discussion

The overall incidence of leukaemia in our study was 11.25 per 100, 000 person years which is very similar to the figure reported by Cancer Research UK which is 11.7 [1]. Similarly, the age and gender distribution of our cases is very similar to those reported by Cancer Research UK [1] and others [5]. CLL and AML were more common in men. The incidence of ALL tended to decrease with increasing deprivation. There has been an increase in the incidence of ALL, CLL and AML over the past 20 years, but whether this represents a true increase or better data recording, or both, is not clear. Our survival figures are also as expected [5] being dismal for AML. Men with CLL had a worse prognosis than women. Prognosis was worse with increasing age at diagnosis in all sub-types. Survival in AML worsened with increasing socio-economic deprivation, a trend not present for other leukaemias. Only the survival for CML has improved with time. The MRC (Medical Research Council, UK) AML trial data show a consistent improvement in survival over time for younger, but not older, patients [6]. The fact that our data does not show an improvement in AML survival over time reflects the overall age distribution of AML, i.e. mostly older people, many of whom are not entered into clinical trials.

The main strength of our study is that the large size of the study population has allowed us to calculate precise up-to-date estimates of the incidence of leukaemia in the UK, and stratify these by age, gender and socio-economic class. Furthermore, the extensive follow-up data contained in this dataset have enabled us to accurately calculate mortality rates. By using THIN data we have also had access to a number of covariates by which to stratify our results. As this was a general population based study, we have had access to more data than would have been the case with a centre-based study, and we have also had access to general population controls. We have therefore been able to conduct a detailed study of high quality.

One potential weakness of this study is the issue of diagnostic validity in the dataset. In other words, do people recorded as having leukaemia really have this condition? It seems to us unlikely that a GP will record a diagnosis of leukaemia unless there is good evidence from secondary care to support this. Furthermore, the age and gender distributions of disease incidence are as expected [1], giving validity to our findings. By having carefully excluded prevalent cases we can be certain that our incidence rates are not spuriously elevated, and that any trends over time have not been masked. The fact that our trends in mortality over time are comparable to those published elsewhere [1] suggests that we have accurately identified incident cases. We acknowledge that a small percentage of cases had non-specific codes and we were therefore unable to classify them into more specific sub-types.

The overall incidence we found is consistent with the crude incidence rate of 11.7 per 100, 000 population published by Cancer Research UK for 2004 [1]. The distribution of age at diagnosis of leukaemia we have shown is also consistent with the findings of others [1,5]. Other studies have shown a similar incidence in ALL in men and women, and a higher incidence in men of both CLL and AML [7,8], in keeping with our results.

The previous studies of leukaemia and socio-economic class have given inconsistent results. Studies prior to the 1980s mainly found higher incidences of leukaemia in higher social classes [7,9,10], in both adults and children. Since the 1980s, however, studies have consistently reported inverse associations with socio-economic class [11]. This apparent change in direction of the association may be explained by differences in study design and/or measures of socio-economic deprivation that have been used over time. Most studies prior to the 1980s were ecological studies whilst after this time most studies were case-control studies and used individual-level measures of income and education, rather than ecological-level indicators of socio-economic status [11]. The more recent studies have therefore classified the socio-economic status of cases more accurately.

Worse survival with increased age at diagnosis is an entirely expected finding and is in keeping with other published data [1]. Our finding of worse survival in men than women with CLL is also consistent with that of others [12]. While men have a lower life-expectancy than women overall, the difference in survival between men and women with CLL may also represent gender differences in disease phenotype, stage of presentation and/or response to treatment, factors which we could not elucidate in this dataset. Studies of the impact of socio-economic class on mortality in leukaemia overall have shown conflicting results [5,7,13]. The results of studies that have investigated the association of socio-economic status and survival in ALL specifically have also been conflicting [11,14]. To our knowledge research into this association in other leukaemia sub-types has not been published more recently than that by Cartwright [7].

## Conclusion

We have demonstrated that general practice data is a valuable resource for leukaemia research. We have been conducted a contemporary population-based incidence and mortality study stratified by age, gender and socio-economic class, which has not been done in the UK before.

We have also shown that AML survival is dismal, and is related to social class. Poorer survival in AML patients from lower socio-economic classes may represent a class bias in treatment offered and/or greater co-morbidity in these patients, and warrants further exploration.

## Competing interests

The authors declare that they have no competing interests.

## Authors' contributions

FB conducted the data management, performed the statistical analyses and drafted the manuscript. EDG contributed to revising the manuscript for intellectual content. CS extracted the relevant data and performed the initial data management. TM reviewed the statistical methods. RH conceived of the study, acquired the data and edited the manuscript. All authors read and approved the final manuscript.

## Funding

This study forms part of a self-funded PhD project for which data was provided by the Division of Epidemiology and Public Health, University of Nottingham.

## Pre-publication history

The pre-publication history for this paper can be accessed here:

http://www.biomedcentral.com/1471-2407/9/252/prepub

## Supplementary Material

Additional file 1 **Crude Incidence Rates per Hundred-Thousand Person-years**. These data show the crude incidence rates of the acute and chronic leukaemias. Click here for file

Additional file 2 **Mutually Adjusted Incidence Rate Ratios**. These data show incidence rate ratios mutually adjusted for all other variables in the table. Click here for file

Additional file 3 **Median Survival by Leukaemia Sub-type**. These data show the median survival in each of the leukaemia subtypes investigated. Click here for file

Additional file 4 **Mutually Adjusted Hazard Ratios for Death**. These data show hazard ratios mutually adjusted for all other variables in the table. Click here for file
