# Supplementary material for: The incidence of and mortality from leukaemias in the UK: a general population-based study
Source: BMC Cancer. 2009 Jul 26;9:252. doi: 10.1186/1471-2407-9-252 (PMC2722672; doi:10.1186/1471-2407-9-252)
Supplement: Additional file 1 — Crude Incidence Rates per Hundred-Thousand Person-years. These data show the crude incidence rates of the acute and chronic leukaemias. [file 1471-2407-9-252-S1.doc]

Table 1: Crude Incidence Rates Per Hundred-Thousand Person-years

|  | **Person-years** | **ALL** | | **CLL** | | **Unspec. Lymph.** | | **AML** | | **CML** | | **Unspec. Myel.** | |
| --- | --- | --- | --- | --- | --- | --- | --- | --- | --- | --- | --- | --- | --- |
|  |  | **Cases** | **IR** | **Cases** | **IR** | **Cases** | **IR** | **Cases** | **IR** | **Cases** | **IR** | **Cases** | **IR** |
| **Overall:** | 36 993 338 | 182 | 0.49 | 1554 | 4.20 | 356 | 0.96 | 602 | 1.63 | 265 | 0.72 | 257 | 0.70 |
|  |  |  |  |  |  |  |  |  |  |  |  |  |  |
| **Gender:** |  |  |  |  |  |  |  |  |  |  |  |  |  |
| **Male** | 18 284 784 | 89 | 0.49 | 914 | 5.00 | 203 | 1.11 | 319 | 1.74 | 138 | 0.75 | 118 | 0.65 |
| **Female** | 18 708 554 | 93 | 0.50 | 640 | 3.42 | 153 | 0.82 | 283 | 1.51 | 137 | 0.73 | 139 | 0.74 |
|  |  |  |  |  |  |  |  |  |  |  |  |  |  |
| **Age at Diagnosis:** |  |  |  |  |  |  |  |  |  |  |  |  |  |
| **<20** | 8 288 518 | 116 | 1.40 | 1 | 0.01 | 20 | 0.24 | 30 | 0.36 | 5 | 0.06 | 7 | 0.08 |
| **20-39** | 10 578 667 | 23 | 0.22 | 6 | 0.06 | 10 | 0.09 | 54 | 0.51 | 23 | 0.22 | 18 | 0.17 |
| **40-59** | 9 995 140 | 18 | 0.18 | 251 | 2.51 | 68 | 0.68 | 123 | 1.23 | 66 | 0.66 | 58 | 0.58 |
| **60-79** | 6 462 905 | 14 | 0.22 | 927 | 14.34 | 185 | 2.86 | 296 | 4.58 | 120 | 1.86 | 115 | 1.78 |
| **80+** | 1 668 108 | 11 | 0.66 | 369 | 22.12 | 73 | 4.38 | 99 | 5.93 | 61 | 3.66 | 59 | 3.54 |
|  |  |  |  |  |  |  |  |  |  |  |  |  |  |
| **Townsend Score:** |  |  |  |  |  |  |  |  |  |  |  |  |  |
| **1** | 8 421 428 | 45 | 0.53 | 356 | 4.22 | 85 | 1.01 | 138 | 1.64 | 65 | 0.77 | 54 | 0.64 |
| **2** | 7 179 869 | 35 | 0.49 | 335 | 4.67 | 82 | 1.14 | 145 | 2.02 | 51 | 0.71 | 66 | 0.92 |
| **3** | 6 952 931 | 36 | 0.52 | 293 | 4.21 | 64 | 0.92 | 111 | 1.60 | 61 | 0.88 | 47 | 0.68 |
| **4** | 6 220 650 | 27 | 0.43 | 269 | 4.32 | 65 | 1.04 | 103 | 1.66 | 40 | 0.64 | 40 | 0.64 |
| **5** | 4 488 547 | 19 | 0.42 | 174 | 3.89 | 35 | 0.78 | 53 | 1.18 | 32 | 0.71 | 31 | 0.69 |
| **No Record** | 3 729 913 | 20 | 0.54 | 127 | 3.40 | 25 | 0.67 | 52 | 1.39 | 26 | 0.70 | 19 | 0.51 |
|  |  |  |  |  |  |  |  |  |  |  |  |  |  |
| **Year of Diagnosis:** |  |  |  |  |  |  |  |  |  |  |  |  |  |
| **1987-1991** | 3 323 812 | 10 | 0.30 | 62 | 1.87 | 29 | 0.87 | 21 | 0.63 | 16 | 0.48 | 13 | 0.39 |
| **1992-1996** | 8 945 190 | 31 | 0.35 | 230 | 2.57 | 102 | 1.14 | 82 | 0.92 | 66 | 0.74 | 49 | 0.55 |
| **1997-2001** | 11 816 760 | 54 | 0.46 | 449 | 3.80 | 104 | 0.88 | 164 | 1.39 | 75 | 0.63 | 88 | 0.74 |
| **2002-2006** | 12 907 576 | 81 | 0.63 | 733 | 5.68 | 113 | 0.88 | 295 | 2.29 | 108 | 0.84 | 103 | 0.80 |
