# Supplementary material for: The incidence of and mortality from leukaemias in the UK: a general population-based study
Source: BMC Cancer. 2009 Jul 26;9:252. doi: 10.1186/1471-2407-9-252 (PMC2722672; doi:10.1186/1471-2407-9-252)
Supplement: Additional file 2 — Mutually Adjusted Incidence Rate Ratios. These data show incidence rate ratios mutually adjusted for all other variables in the table. [file 1471-2407-9-252-S2.doc]

Table 2: Mutually Adjusted Incidence Rate Ratios (95% Confidence Intervals)

|  | **ALL** | **CLL** | **Unspec. Lymph.** | **AML** | **CML** | **Unspec. Myel.** |
| --- | --- | --- | --- | --- | --- | --- |
| **Gender:** |  |  |  |  |  |  |
| **Female** | 1.05(0.78-1.42) p=0.73 | 0.55(0.49-0.61) p<0.001 | 0.62(0.50-0.77) p<0.001 | 0.78(0.66-0.92) p<0.001 | 0.84(0.66-1.08) p=0.17 | 0.96(0.75-1.23) p=0.75 |
| **Diagnosis Age:** |  |  |  |  |  |  |
| **<20** | 1 | } | 1 | 1 | 1 | 1 |
| **20-39** | 0.15(0.09-0.23) | } 1^!^ | 0.40 (0.19-0.85) | 1.39(0.88-2.20) | 3.32(1.25-8.82) | 2.05(0.86-4.91) |
| **40-59** | 0.11(0.06-0.18) | 74.90 (33.32-168.39) | 2.73(1.66-4.50) | 3.28(2.19-4.93) | 10.41(4.19-25.89) | 6.83(3.12-14.97) |
| **60-79** | 0.16(0.09-0.27) | 433.89(194.39-968.48) | 11.74(7.40-18.64) | 12.34(8.42-18.11) | 29.88(12.20-73.15) | 20.35(9.48-43.69) |
| **80+** | 0.47(0.25-0.87)*p<0.001 | 731.21(326.22-1638.97)*p<0.001 | 19.87(12.08-32.68)*p<0.001 | 16.30(10.72-24.77)*p<0.001 | 62.67(25.15-156.17)*p<0.001 | 41.94(19.13-91.96) *p<0.001 |
| **Townsend Score:** |  |  |  |  |  |  |
| **1** | 1 | 1 | 1 | 1 | 1 | 1 |
| **2** | 0.92(0.59-1.44) | 1.02(0.87-1.19) | 1.03(0.76-1.40) | 1.16(0.91-1.48) | 0.81(0.56-1.18) | 1.32(0.92-1.89) |
| **3** | 0.92(0.59-1.43) | 0.97(0.83-1.14) | 0.89(0.64-1.23) | 0.94(0.72-1.21) | 1.09(0.77-1.55) | 1.00(0.69-1.49) |
| **4** | 0.79(0.49-1.27) | 1.04(0.88-1.22) | 1.02(0.74-1.42) | 1.03(0.79-1.34) | 0.75(0.50-1.21) | 0.97(0.64-1.47) |
| **5** | 0.70(0.41-1.22)*p=0.15 | 0.97(0.80-1.17)*p=0.89 | 0.78(0.53-1.17)*p=0.89 | 0.77(0.55-1.06)*p=0.89 | 0.90(0.58-1.39)*p=0.88 | 1.11(0.72-1.73) *p=0.81 |
| **No Record** | 1.00(0.58-1.71) | 0.93(0.76-1.14) | 0.64(0.41-1.01) | 0.92(0.66-1.29) | 0.92(0.58-1.45) | 0.78(0.46-1.33) |
| **Diagnosis Year:** |  |  |  |  |  |  |
| **1987-1991** | 1 | 1 | 1 | 1 | 1 | 1 |
| **1992-1996** | 1.14(0.56-2.33) | 1.34(1.01-1.77) | 1.25(0.83-1.90) | 1.43(0.88-2.31) | 1.50(0.87-2.60) | 1.35(0.73-2.49) |
| **1997-2001** | 1.48(0.75-2.91) | 1.96(1.50-2.56) | 0.96(0.63-1.44) | 2.15(1.37-3.39) | 1.29(0.75-2.21) | 1.82(1.01-3.26) |
| **2002-2006** | 2.01(1.04-3.88)*p<0.001 | 2.87(2.22-3.73)*p<0.001 | 0.93(0.62-1.40)*p=0.11 | 3.50(2.25-5.46)*p<0.001 | 1.67(0.99-2.83)*p=0.10 | 1.92(1.08-3.42) *p=0.01 |

^!^ Baseline age category for CLL is age<40.

*p=test for trend across ordered categories.

Records with missing Townsend Scores were not included in trend analysis for Townsend Score.
