# Supplementary material for: The incidence of and mortality from leukaemias in the UK: a general population-based study
Source: BMC Cancer. 2009 Jul 26;9:252. doi: 10.1186/1471-2407-9-252 (PMC2722672; doi:10.1186/1471-2407-9-252)
Supplement: Additional file 3 — Median Survival by Leukaemia Sub-type. These data show the median survival in each of the leukaemia subtypes investigated. [file 1471-2407-9-252-S3.doc]

Table 3: Median Survival by Leukaemia Subtype

| **Subtype** | **Number of Cases** | **Median Survival**  **(years)** | **95% Conf. Interval** |
| --- | --- | --- | --- |
| **ALL*** | 180 | . | . |
| **CLL** | 1 549 | 9.53 | 8.20 - 10.18 |
| **Unspecified Lymphoid** | 352 | 10.05 | 6.83 - . |
| **AML** | 593 | 0.79 | 0.64 - 1.00 |
| **CML** | 274 | 5.06 | 3.67 - 6.07 |
| **Unspecified Myeloid** | 250 | 0.81 | 0.59 - 1.39 |
| **OVERALL** | **3 198** | **6.58** | **6.16 - 7.46** |

*5 yr survival for ALL was 69%
