# Supplementary material for: The incidence of and mortality from leukaemias in the UK: a general population-based study
Source: BMC Cancer. 2009 Jul 26;9:252. doi: 10.1186/1471-2407-9-252 (PMC2722672; doi:10.1186/1471-2407-9-252)
Supplement: Additional file 4 — Mutually Adjusted Hazard Ratios for Death. These data show hazard ratios mutually adjusted for all other variables in the table. [file 1471-2407-9-252-S4.doc]

Table 4: Mutually Adjusted Hazard Ratios for Death (95% Confidence Intervals)

|  | **ALL** | **CLL** | **Unspec.Lymph.** | **AML** | **CML** | **Unspec.Myel.** |
| --- | --- | --- | --- | --- | --- | --- |
| **Gender:** |  |  |  |  |  |  |
| **Female** | 1.29(0.70-2.37) p=0.42 | 0.58(0.48-0.71) p<0.001 | 0.58(0.40-0.82) p<0.001 | 0.88(0.71-1.09) p=0.25 | 0.87(0.60-1.26) p=0.46 | 1.00(0.73-1.38) p=1.00 |
|  |  |  |  |  |  |  |
| **Diagnosis Age:** |  |  |  |  |  |  |
| **<20** | 1 | } | 1 | 1 | } | 1 |
| **20-39** | 1.14(0.37-3.48) | } 1^!^ | 3.96(0.35-44.27) | 1.68(0.60-4.73) | } 1^!^ | 0.83(0.16-4.21) |
| **40-59** | 5.03(2.21-11.40) | 0.34(0.08-1.45) | 6.34(0.84-47.92) | 4.56(1.83-11.36) | 0.99(0.38-2.56) | 1.28(0.30-5.51) |
| **60-79** | 20.11(8.11-49.84) | 1.04(0.26-4.21) | 11.23(1.55-81.22) | 10.22(4.18-24.96) | 2.44(1.04-5.73) | 3.51(0.84-14.58) |
| **80+** | 6.07(1.89-19.48)*p<0.001 | 2.80(0.69-11.38) *p<0.001 | 29.87(4.08-218.60) *p<0.001 | 23.33(9.30-58.52)*p<0.001 | 7.26(2.93-17.97) *p<0.001 | 6.26(1.50-26.23) *p<0.001 |
|  |  |  |  |  |  |  |
| **Townsend Score:** |  |  |  |  |  |  |
| **1** | 1 | 1 | 1 | 1 | 1 | 1 |
| **2** | 0.96(0.39-2.35) | 1.09(0.82-1.45) | 0.72(0.42-1.24) | 1.17(0.86-1.60) | 0.58(0.31-1.09) | 1.04(0.66-1.64) |
| **3** | 1.06(0.43-2.59) | 1.25(0.94-1.67) | 2.14(1.31-3.52) | 1.33(0.97-1.84) | 0.90(0.53-1.54) | 0.83(0.50-1.38) |
| **4** | 0.82(0.31-2.17) | 1.13(0.84-1.53) | 0.67(0.38-1.20) | 1.34(0.96-1.86) | 0.90(0.48-1.69) | 0.75(0.44-1.26) |
| **5** | 0.20(0.05-0.87)*p=0.08 | 1.14(0.80-1.61) *p=0.31 | 0.86(0.46-1.61) *p=0.77 | 1.48(0.97-2.27)*p=0.03 | 0.74(0.38-1.44)*p=0.80 | 0.85(0.47-1.53)*p=0.26 |
| **No Record** | 0.74(0.25-2.16) | 1.82(1.29-2.57) | 1.20(0.60-2.42) | 1.04(0.68-1.60) | 1.15(0.59-2.24) | 0.66(0.29-1.52) |
|  |  |  |  |  |  |  |
| **Diagnosis Year:** |  |  |  |  |  |  |
| **1987-1991** | 1 | 1 | 1 | 1 | 1 | 1 |
| **1992-1996** | 3.12(0.66-14.70) | 0.85(0.59-1.23) | 0.85(0.47-1.51) | 0.60(0.34-1.05) | 0.72(0.37-1.42) | 1.32(0.53-3.30) |
| **1997-2001** | 3.87(0.87-17.16) | 0.98(0.68-1.41) | 0.90(0.48-1.67) | 0.53(0.31-0.91) | 0.77(0.39-1.52) | 1.14(0.47-2.74) |
| **2002-2006** | 2.95(0.68-12.90)*p=0.79 | 1.05(0.72-1.54) *p=0.56 | 1.22(0.64-2.31) *p=0.76 | 0.55(0.32-0.92)*p=0.84 | 0.48(0.24-0.97)*p=0.05 | 1.09(0.45-2.62) p=0.26 |

^!^ Baseline age category for CLL and CML is age<40.

*p=test for trend across ordered categories.

Records with missing Townsend Scores were not included in trend analysis for Townsend Score.
